# Intraoperative Identification and Stimulation of the Ansa Cervicalis Nerve Plexus

**DOI:** 10.1002/ohn.70079

**Published:** 2025-12-07

**Authors:** Connie C. Ma, Kyle Mannion, Michael C. Topf, Sarah L. Rohde, Alexander J. Langerman, James L. Netterviell, Robert Sinard, David Zealear, Yike Li, Alan. R. Schwartz, Silvana Bellotto, Carol LeeAnn Wells, Katherine E. Estes, David T. Kent

**Affiliations:** ^1^ Department of Otolaryngology–Head & Neck Surgery Vanderbilt University Medical Center Nashville Tennessee USA; ^2^ Department of Otorhinolaryngology University of Pennsylvania Perelman School of Medicine Philadelphia Pennsylvania USA; ^3^ Universidad Peruana Cayetano Heredia School of Medicine Lima Peru; ^4^ Hospital Vila Nova Star São Paulo Brazil

**Keywords:** ansa cervicalis, neuromodulation, neurostimulation, obstructive sleep apnea

## Abstract

**Objective:**

Quantify the anatomic variation of the ansa cervicalis and evaluate neurostimulation of the infrahyoid musculature.

**Study Design:**

Intraoperative physiology study.

**Setting:**

Tertiary referral center.

**Methods:**

Adult patients undergoing lateral neck dissection including level IV for head and neck cancer were recruited. Ansa cervicalis plexus branches were documented during surgical dissection. In a subset of participants, an electrode was placed on branches of the common sternothyroid trunk innervating the sternothyroid and sternohyoid muscles. Hyolaryngeal excursion with neurostimulation was recorded.

**Results:**

Measurements were collected from 39 of 50 participants. Reasons for intraoperative exclusion included significant radiation fibrosis (n = 4), nodal disease burden (n = 3), and surgeon preference (n = 4). The mean lengths of the common sternothyroid trunk and sternothyroid branch were 37.6 ± 15.0 mm and 20.4 ± 8.1 mm, respectively. Their respective mean diameters were 2.1 ± 0.7 mm and 1.5 ± 0.5 mm. The distance from sternothyroid branch muscle insertion to the sternum varied substantially (12.8 ± 14.8 mm). Nine patients underwent neurostimulation of the common sternothyroid trunk. The amplitude of first observed muscle contraction was 0.35 ± 0.18 mA and maximal was 0.57 ± 0.40 mA, during which the hyolaryngeal complex descended by 13.6 ± 4.6 mm. In patients with anatomy amenable to neurostimulation of other nerve branches, stimulation of the common sternothyroid trunk produced the greatest hyolaryngeal descent (*P* < .05).

**Conclusion:**

The minor variability observed in ansa cervicalis nerve diameter across patients and branches supports the feasibility of a standardized electrode design for an implantable neurostimulation device. Stimulation of the common sternothyroid trunk resulted in the greatest hyolaryngeal descent, highlighting its value as a potential neuromodulation target.

Obstructive sleep apnea (OSA) is a highly prevalent sleep disorder characterized by repetitive upper airway collapse that has well established health consequences and impact on all‐cause mortality.^1^, ^2^ Continuous positive airway pressure is the gold standard for disease management, however, its therapeutic effect is limited by suboptimal patient adherence to therapy.^3^ Several surgical options aim to address upper airway obstruction and collapse, including traditional procedures that statically alter upper airway anatomy and newer dynamic therapies such as hypoglossal nerve stimulation (HNS). However, only a subset of patients with moderate‐to‐severe obstructive sleep apnea are candidates for this therapy and approximately one third of patients respond incompletely to treatment.^4^, ^5^

The ansa cervicalis nerve plexus has previously been described as a novel neurostimulation target for the treatment of OSA. Whereas genioglossus contraction with HNS pulls pharyngeal structures ventrally, infrahyoid strap muscle contraction with ansa cervicalis stimulation (ACS) is hypothesized to stabilize the upper airway via multiple mechanical effects caused by hyolaryngeal descent.^6^, ^7^, ^8^ Several pilot studies have shown that stimulation of the common sternothyroid trunk (CT) of the ansa cervicalis increases upper airway patency in patients with OSA.^9^, ^10^, ^11^, ^12^ Combining ACS and HNS may yield greater decreases in airway collapsibility than either modality in isolation.^10^, ^11^, ^12^

The omo‐ and sternohyoid muscles receive dual innervation from inconstant anatomic origins, but cadaveric studies indicate that the sternothyroid muscle and the inferior belly of the sternohyoid muscle are reliably innervated by a single CT branch, making it a compelling neurostimulation target.^13^, ^14^ Nevertheless, anatomic details of the CT, including its length, diameter, and features of its tributaries remain poorly defined. In the current study, we quantified the natural anatomic variation of the CT in vivo during neck dissection operations, with specific attention to the branch to the sternothyroid muscle. We additionally evaluated neurostimulation responses and stimulation current thresholds for sternothyroid and inferior sternohyoid muscle contraction.

## Methods

This study was approved by the Vanderbilt University Medical Center Institutional Review Board (IRB #221998). Participants were recruited from a group of patients with head and neck cancer scheduled to undergo selective lateral neck dissection including level IV as part of their standard of care. Patients with a history of prior neck surgery resulting in potential transection of the infrahyoid strap muscles (eg, thyroidectomy) were excluded. Demographic data and relevant clinical data were collected for each patient.

**Figure 1:**
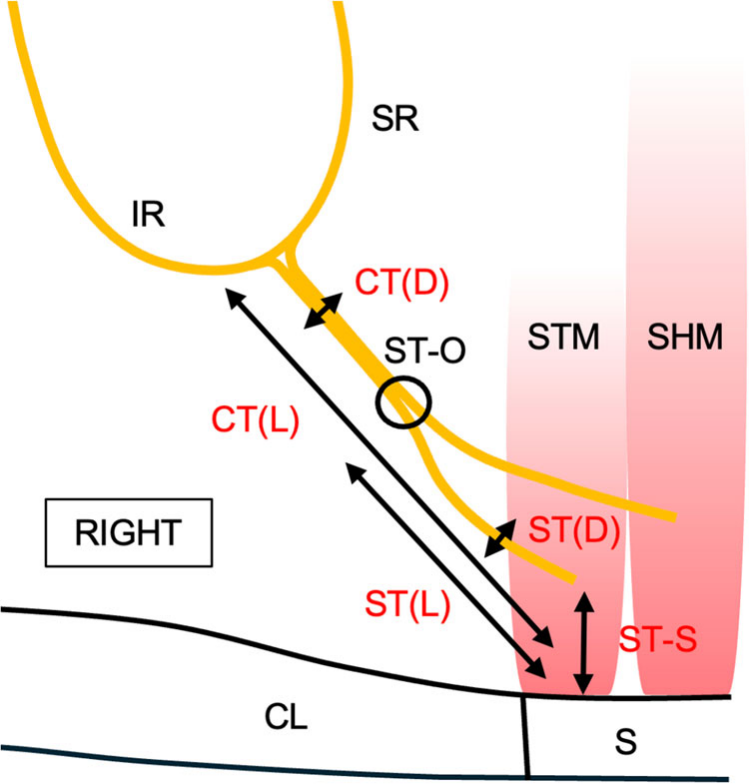
A schematic of recorded anatomic measurements. CT, common sternothyroid trunk; CL, clavicle; D, diameter; IR, inferior root of ansa cervicalis; L, length; S, sternum; SHM, sternohyoid muscle; SR, superior root of ansa cervicalis; ST, sternothyroid branch; STM, sternothyroid muscle; ST‐O, ST origin.

After signing informed consent, participants were administered general anesthesia and standard oncologic surgery was initiated. During routine level IV neck dissection, branches of the ansa cervicalis nerve plexus were identified and preserved. Following completion of nodal dissection, the course of the CT was isolated between its origin at the loop of the ansa cervicalis to its distal muscle insertions. Anatomic measurements including the length and diameter of the CT and the sternothyroid branch, as well as the distance from the sternothyroid muscle insertion to the sternum, were recorded (Figure 1). If bilateral neck dissections were performed, the distance between sternothyroid branch origins was documented. Representative photographs and video were additionally collected.

In a subset of 10 consecutive patients, 3‐contact platinum‐iridium spiral cuff electrodes (AirRay Spiral Cuff Electrode, 2 mm diameter; CorTec Neuro) were used to investigate the mechanical effects of different ACS modalities. The electrodes were connected to a neurostimulation unit (Digitimer DS7A; Digitimer Ltd) in a bipolar configuration using a center and outer electrode contact. Patients with a history of head and neck radiation therapy were excluded from this portion of the study. For these patients, pharmacologic neuromuscular blockade was withheld after intubation. Following collection of anatomic measurements, a single sterile electrode was connected to the neurostimulation unit, brought into the surgical field, and placed on the CT. ACS (square‐wave pulses, 200 μs, 30 Hz) amperage was titrated upward in 0.01 mA increments until infrahyoid muscle twitch was observed with single pulse stimulation. Stimulation was then applied in approximately 2 to 3 seconds bursts and amperage was increased until maximal muscle contraction was observed, meaning that associated hyolaryngeal structure descent did not increase further. The amperage corresponding to initial and maximal muscle contraction was documented, as were the degree of associated hyoid bone and thyroid notch descent. Photographs and video of each neurostimulation procedure were recorded. In patients with adequate sternothyroid and/or inferior sternohyoid branch length (approximately 15 mm or greater), the cuff electrode was moved and neurostimulation procedures were repeated. The cuff electrode was then removed from the surgical field, completing the research portion of the surgical procedure.

Statistical analysis was conducted using GraphPad Prism, version 10.3.0 (GraphPad Prism Software, Inc.). Categorical variables were summarized with incidence counts and frequency percentages, and continuous variables were expressed as mean and standard deviation (SD). One‐way analysis of variance tests or the paired‐sign test were performed across continuous variables. Statistical significance was determined at the threshold of 0.05.

## Results

**Figure 2:**
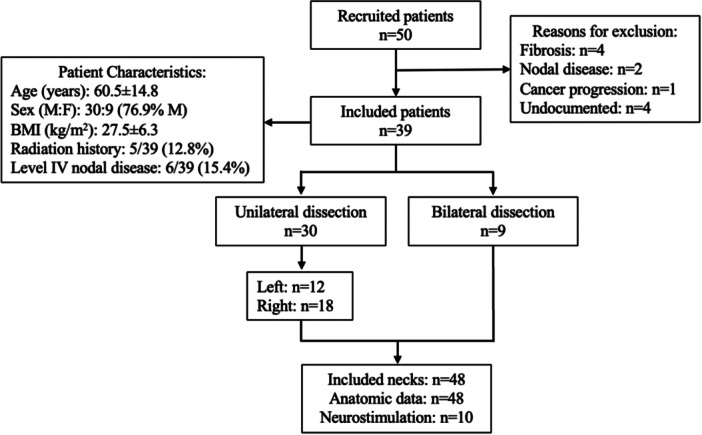
Flowchart of the study population.

Anatomic data were collected from 39 of 50 eligible and consented participants between April 2023 and December 2024. Reasons for lack of intraoperative data collection included significant radiation fibrosis (n = 4), oncologic disease burden preventing preservation of ansa cervicalis plexus structures for research procedures (n = 3), or surgeon preference to proceed with the oncologic resection (n = 4). Participants were primarily middle‐aged or elderly (60.5 ± 14.8 years) males (n = 30, 76.9%). Five participants (12.8%) had history of prior radiation therapy to the neck, and 6 patients (15.4%) had radiographic evidence of level IV nodal disease. Most patients underwent unilateral neck dissection (n = 30) for a variety of different primary cancer pathologies; 18 cases were right neck dissections, and 12 cases were left neck dissections. Nine participants underwent bilateral neck dissection. In total, anatomic data were collected from 48 necks across 39 participants (Figure 2). No adverse events were encountered through the course of the study.

On average, the length of the CT was 37.6 ± 15.0 mm and the sternothyroid branch was 20.4 ± 8.1 mm. There was no significant difference in CT or sternothyroid branch length between the right and left sides (38.7 ± 12.9 mm vs 36.3 ± 17.6 mm, *P* = .586; 21.5 ± 8.6 mm vs 19.0 ± 7.5 mm, *P* = .283; respectively). The distance from sternothyroid branch muscle insertion to the sternum was highly variable, with an average distance of 12.8 ± 14.8 mm (range: 0‐60 mm). The average diameter of the CT was 2.1 ± 0.7 mm and the sternothyroid branch was 1.5 ± 0.5 mm. The CT and sternothyroid branch nerves ranged in diameter of from 1.0‐4.0 mm. The diameter of the CT was significantly smaller on the right compared to the left side (2.0 ± 0.5 mm vs 2.4 ± 0.8 mm, *P* = .023) (Table 1). In patients who underwent bilateral neck dissection, anatomic measurements of the right and left sides were statistically similar (Table 2).

**Table 1:** Summary Anatomic Data

	Overall (n = 48)	Right (n = 27)	Left (n = 21)	*P*
CT length CT(L)	37.6 ± 15.0 (10.0‐80.0)	38.7 ± 12.9 (18.8‐65.0)	36.3 ± 17.6 (10.0‐80.0)	.586
CT diameter CT(D)	2.1 ± 0.7 (1.0‐4.0)	2.0 ± 0.5 (1.0‐3.5)	2.4 ± 0.8 (1.0‐4.0)	.023
ST length ST(L)	20.4 ± 8.1 (7.0‐44.0)	21.5 ± 8.6 (10.0‐44.0)	19.0 ± 7.5 (7.0‐34.0)	.283
ST diameter ST(D)	1.5 ± 0.5 (0.8‐3.0)	1.5 ± 0.5 (0.8‐2.5)	1.4 ± 0.6 (1.0‐3.0)	.497
ST insertion to sternum (ST‐S)	12.8 ± 14.8 (0.0‐60.0)	10.3 ± 13.9 (0.0‐60.0)	16.0 ± 15.6 (0.0‐50.0)	.188
Distance between ST origins (Right ST‐O to Left ST‐O)	70.2 ± 4.0 (65.0‐75.0, n = 5)			

All measurements are documented in millimeters.

Abbreviations: D, diameter; L, length; S, sternum; ST‐O, ST origin.

**Table 2:** Bilateral Surgical Dissection Anatomic Data

	CT(L)		CT(D)		ST(L)		ST(D)		ST‐S	
Patient	Right	Left	Right	Left	Right	Left	Right	Left	Right	Left
1	47	33	2	4	13	28	1.5	2	0	0
2	30	35	1.5	2	10	20	1	1	30	25
3	65	80	2	3	22	24	1	1	5	12
4	46	41	1.5	1	28	25	1	1	0	0
5	30	30	2	1.5	20	20	2	2	15	20
6	20	12	2	2	15	10	1	1	15	25
7	35	25	2	3	12	10	1	1	5	5
8	27	29	2	2	13	20	2	2	15	13
9	45	55	2.5	2	15	27	1.5	1.25	15	20
	*P* = .866		*P* = .282		*P* = .135		*P* = .681		*P* = .202	

Nine patients underwent bilateral surgical dissection. In these patients, anatomic measurements of the right and left sides were statistically similar. All measurements are documented in millimeters.

**Figure 3:**
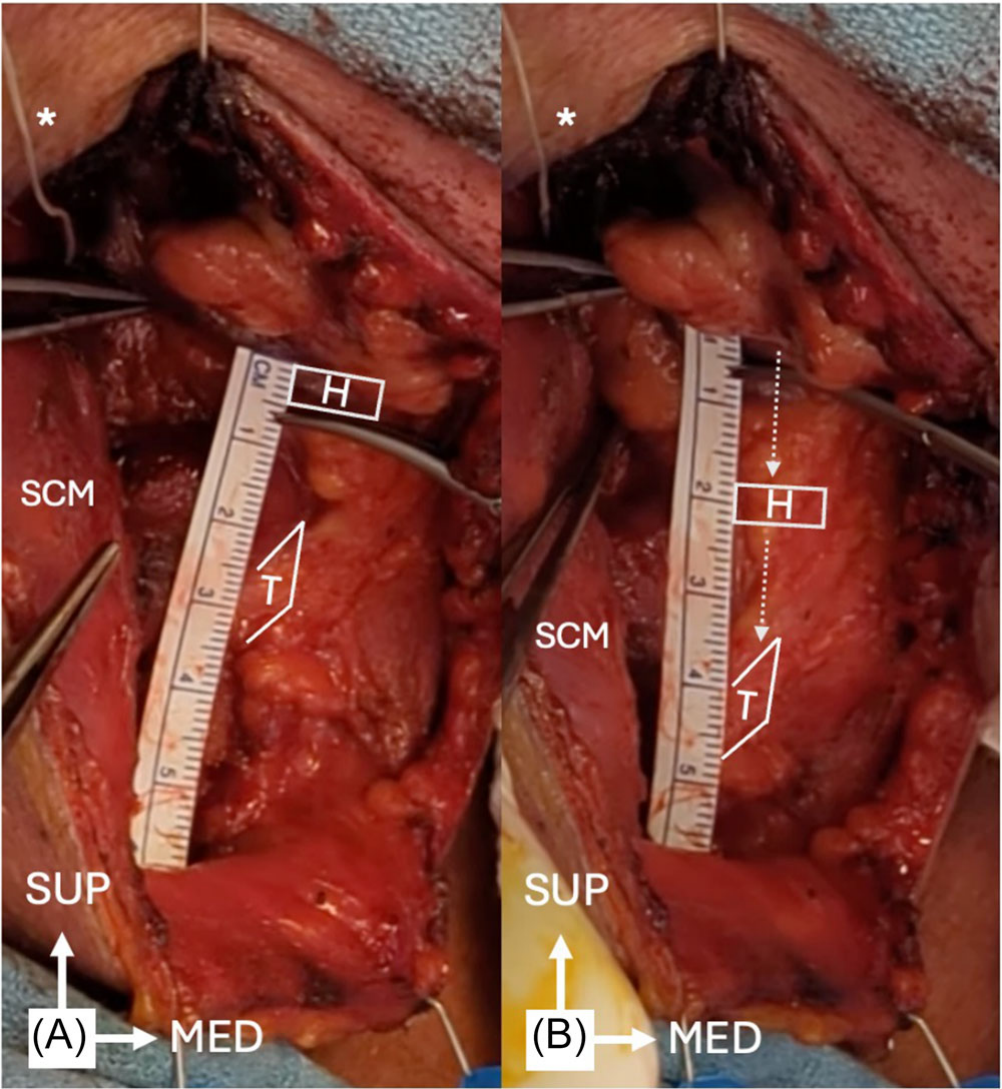
Neurostimulation of the right common sternothyroid trunk of the ansa cervicalis. *, cuff electrode wire. MED, medial; SCM, sternocleidomastoid muscle; SUP, superior. (A) H, hyoid bone; T, thyroid cartilage. (B) 18 mm hyolaryngeal complex descent (dashed arrows) with ACS (0.7 mA).

Ten participants additionally underwent neurostimulation procedures (Figure 3 and Video 1). Testing was aborted in one participant who had inadvertently received intraoperative neuromuscular blockade, resulting in lack of muscle contraction with ACS. In total, CT neurostimulation data were collected from 10 necks across 9 participants. The average amplitude of first observed muscle contraction was 0.35 ± 0.18 mA and maximal contraction was 0.57 ± 0.40 mA. Average descent of the hyolaryngeal complex with maximal contraction was 13.6 ± 4.6 mm (Table 3). Hyoid bone and thyroid notch descent were observed to be identical during ACS of the CT, without appreciable differences in the descent of the separate structures. In patients with anatomy amenable to separate stimulation of the sternothyroid (n = 5) and/or inferior sternohyoid nerve (n = 5) branches, CT stimulation produced greater descent of the hyoid bone (12.5 ± 4.8 mm) than either isolated sternothyroid (7.2 ± 5.3 mm; *P* < .05) or sternohyoid branch stimulation (6.2 ± 2.9 mm; *P* < .05). CT stimulation produced greater descent of the thyroid cartilage (12.5 ± 4.8 mm) than isolated sternohyoid branch stimulation (5.4 ± 2.8 mm; *P* < .05), but there was no statistically significant difference from isolated sternothyroid branch stimulation (8.2 ± 4.4 mm; *P* = .17).

**Table 3:** Neurostimulation Data

Neck (n)	Branch	Initial muscle contraction (mA)	Maximal muscle contraction (mA)	Hyoid bone descent (mm)	Thyroid cartilage descent (mm)
1	CT	0.40	0.65	18	18
2	CT	0.70	1.50	10	10
3	CT	0.14	0.20	13	13
4	CT	0.14	0.50	16	16
	ST branch	0.19	0.50	12	12
	SH branch	0.13	0.40	10	10
5	CT	0.42	0.90	15	15
	ST branch	0.20	0.45	10	10
6	CT	0.26	0.55	10	10
	ST branch	0.51	1.00	1	1
	SH branch	0.18	0.25	8	4
7	CT	0.30	0.50	18	18
	SH branch	0.80	0.80	4	4
8	CT	0.40	1.20	5	5
	ST branch	0.20	0.70	2	7
	SH branch	0.30	0.80	3	3
9	CT	0.55	0.55	11	11
	ST branch	0.16	0.16	11	11
	SH branch	0.48	0.48	6	6
10	CT	0.20	0.20	20	20
Total	CT	0.35 ± 0.18	0.57 ± 0.40	13.6 ± 4.6	13.6 ± 4.6
	ST branch	0.25 ± 0.15	0.56 ± 0.31	8.2 ± 4.4	7.8 ± 5.0
	SH branch	0.38 ± 0.27	0.55 ± 0.25	5.3 ± 2.9	5.8 ± 3.1

Individual and summary data detailing amperage of initial and maximal muscle contraction and distance of hyoid bone descent with stimulation of the common ST trunk, ST branch, and SH branch.

## Discussion

This in vivo cross‐sectional study quantified anatomic characteristics of the common trunk of the ansa cervicalis nerve plexus and its tributaries. The CT was reliably identified in all participants in whom oncologic burden or prior radiation fibrosis were not too excessive. ACS of the CT in a subset of nine participants generated 1 to 2 cm descent of hyolaryngeal structures across a relatively narrow range of tested amperages. It appeared to generate substantially more hyoid bone descent than stimulation of isolated tributary nerves to the sternothyroid or sternohyoid muscles, although the substantial difference in thyroid cartilage descent between CT and isolated sternothyroid branch activation was not statistically significant. Taken together, the results suggest that the CT is a viable neurostimulation target for the potential treatment of obstructive sleep apnea.^9^, ^10^, ^11^, ^12^, ^15^, ^16^

Prior cadaveric studies have documented that the ansa cervicalis is anatomically inconstant, with frequent variation in contributing spinal rootlets, anastomosis, and innervation patterns.^13^, ^14^ The superior root is more variable than the inferior root, with a variety of documented contributions to the superior bellies of the omo‐ and sternohyoid muscles. It can also contain a high degree of retrograde nerve fibers passing rostrally from the inferior root to innervate suprahyoid musculature.^14^ Anastomoses with the vagus and phrenic nerves have been documented, although the functional implications of these connections remain unknown.^13^, ^17^, ^18^ Despite these variations in the superior and inferior roots, the CT is documented to be comparatively invariant, with a single or a few closely aligned efferent fibers descending from the loop of the ansa cervicalis to innervate the infrahyoid strap muscles. These qualities and our findings suggest that: (1) reliable surgical identification of the CT is feasible and (2) ACS of the CT may provide more robust hyolaryngeal descent than isolated tributary branches.

In this study, the CT had an average length of 37.6 ± 15.0 mm, comparable with previous reports of 3 to 4 cm, providing ample length for surgical localization and placement of a neurostimulation electrode.^19^, ^20^ The average diameter of the CT in this in vivo study was 2.1 ± 0.7 mm, larger than previous reports of 0.5 to 0.9 mm,^19^, ^20^ which we surmise may be due to loss of tissue turgor in cadaveric preparation. The diameter of the CT was significantly smaller between the right and left sides (2.0 ± 0.5 mm vs 2.4 ± 0.8 mm, *P* = .023), which may have implications for the amount of current necessary for target muscle activation. Despite prior reports of a narrow range for sternothyroid nerve innervation of the muscle 10 to 20 mm above the sternum,^15^ the average distance in the present study varied substantially from 0 to 60 mm, with no significant difference between the right and left sides. Variation in vertical height to muscle insertion may need to be considered by clinicians or engineering teams when designing surgical techniques and devices for ACS.

Neurostimulation of the CT in 9 participants resulted in a robust muscle response, with hyolaryngeal movement increasing to 1 to 2 cm over an average range of less than 1 mA. In the 6 study patients with anatomy also amenable to neurostimulation of the sternothyroid and sternohyoid nerve branches, stimulation of CT appeared to produce almost double the degree of hyolaryngeal descent. Although CT stimulation generally resulted in greater thyroid cartilage depression, the difference was not statistically different. We hypothesize this may be due to the observed variation in response across the small sample size, although it may also reflect that the single nerve branch innervating the sternothyroid muscle may generate muscle contraction comparable to the more proximate CT, unlike the dual innervation of the sternohyoid muscle. Nevertheless, the low amplitudes for maximal hyolaryngeal movement in this study may not reflect those eventually observed with chronic ACS implants for several reasons. First, the electrode used in this study was large relative to the target nerve. Nerve activation potentials are a function of charge density, and the surface area of the investigated electrode likely created a larger field of charge than would be used in a chronically implanted device. Second, hyolaryngeal movements were assessed during open surgical dissection, when the investigated structures were free of contact with surrounding neck structures that might have otherwise resisted movement. Third, it remains unclear what degree of hyolaryngeal excursion is necessary for optimal therapeutic effect, or whether a patient would be able to sleep comfortably with maximal hyolaryngeal descent. Fourth, only unilateral ACS was tested in this study, and it is unclear whether bilateral ACS would yield different degrees of structural movement. Anecdotally, in percutaneous fine‐wire electrode studies of ACS we have observed a greater decrease in airway collapsibility with bilateral ACS, as unilateral ACS appears to cause the airway to tilt to the ipsilateral side instead of a broader, more symmetrical effect. Further study is needed to ascertain the optimal parameters for ACS in a chronic implant.

We anticipate the findings of this study will inform the potential development of implantable ACS devices. A key strength of this study is the collection of data in the intraoperative setting, which allowed for direct, in vivo observations of anatomic measurements and neuromodulation responses. While we expect these anatomic findings to be generalizable to the larger population of patients with OSA, it is important to acknowledge some of the unique considerations in the head and neck cancer cohort, such as radiation change and nodal disease, that may have contributed to measurement variability.

## Author Contributions

**Connie C. Ma**: conduct, analysis, presentation; **Kyle Mannion**: conduct, presentation; **Michael C. Topf**: conduct, presentation; **Sarah L. Rohde**: conduct, presentation; **Alexander J. Langerman**: conduct, presentation; **James L. Netterville**: conduct, presentation; **Robert Sinard**: conduct, presentation; **David Zealear**: design, analysis, presentation; **Yike Li**: design, analysis, presentation; **Alan. R. Schwartz**: design, analysis, presentation; **Silvana Bellotto**: design, analysis, presentation; **Carol LeeAnn Wells**: conduct, analysis, presentation; **Katherine E. Estes**: conduct, analysis, presentation; **David T. Kent**: design, conduct, analysis, presentation.

## Disclosures

### Competing interests

ARS is a scientific advisor for Apnimed, HuMannity Foundation, Itimar/Zoll, LivaNova, Lunair, Nyxoah SA, Periodic Breathing LLC, Respicardia/Zoll, Restera, Inc., and Takeda. DTK has received research support from Restera, Inc., Inspire Medical Systems, Inc., and Nyxoah SA, is a consultant to Nyxoah SA and Restera, Inc., a scientific advisory board member with Nyxoah SA, and an inventor on patents pending licensed by Nyxoah SA that are relevant to ansa cervicalis stimulation.

### Funding source

This work was enabled by financial support from Nyxoah SA. The funder was not involved in the study design, collection, analysis, interpretation of data, the writing of this article, or the decision to submit it for publication.

This work was presented at the 2025 American Thoracic Society Meeting; May 16‐21, 2025; San Francisco, California

## Supporting information

Video 1. An illustrative video example of ansa cervicalis neurostimulation. *=cuff electrode on the common sternothyroid trunk; SUP=superior; MED=medial; H=hyoid bone; T=thyroid cartilage; M = mandible; SCM = sternocleidomastoid muscle.
